# Addressing unmet mental health needs of older adults in Turbo, Colombia: a multi-component psychosocial intervention feasibility study

**DOI:** 10.1186/s12939-025-02381-x

**Published:** 2025-01-20

**Authors:** Clarissa Giebel, Erika Montoya, Gabriel Saldarriaga, Thais Caprioli, Mark Gabbay, Danicza Martinez, Jessica Rua, Maria Isabel Zuluaga

**Affiliations:** 1 Department of Primary Care & Mental Health, University of Liverpool, Liverpool, UK; 2 NIHR Applied Research Collaboration North West Coast, Liverpool, UK; 3 National School of Public Health, University of Antioquia, Medellín, Colombia; 4 Fundación Instituto Neurológico de Colombia, Medellín, Colombia

**Keywords:** Mental health, Ageing, Community

## Abstract

**Background:**

Older adults have lived through extreme and stressful live events in Colombia, including during the armed conflict. Without adequate mental health resources in place, the aim of this study was to feasibility test a co-produced community-integrated intervention for older adults to improve their mental health and well-being in Turbo, Colombia.

**Methods:**

Based on a systematic review and meta-analysis of community-based mental health interventions for older adults in LMICs, qualitative interviews with older adults and local stakeholders, as well as a mental health needs assessment survey of the local older adult population in Turbo, Colombia, we consulted older adults in the region to co-produce a community-based intervention. The co-produced intervention ran for three months in 2023, with two sessions provided per week in a community centre (26 sessions in total). The multi-component intervention included social engagement, educational interventions, physical activities, and peer support. Older adults were recruited from the local community. Twelve participants were interviewed about their experiences of the intervention and its feasibility.

**Results:**

Eighteen older adults participated in the intervention, with 13 completing the 12 weeks. Attendance rate was high, with 10 participants attending between 90 to 100% of all 26 sessions. Qualitative interviews with 12 participants showed that participants valued the intervention and the activities it offered, that the intervention was feasible, and expressed a keen interest for the intervention to be continued.

**Conclusions:**

This co-produced and evidence-based intervention for older victims of ‘La Violencia’ in Colombia has the potential to provide affordable, acceptable and relevant community-based resources supporting mental health and wellbeing within the community; providing care and support with trained facilitation. Normally, this group would not be able to access services to address their social and psychological isolation and distress. In light of limited mental health support across LMICs, this intervention could provide mental health for older adults in other communities in Colombia and elsewhere developed through co-production, cultural adaptation, subject to further evaluation.

**Supplementary Information:**

The online version contains supplementary material available at 10.1186/s12939-025-02381-x.

## Introduction

Globally, the number and proportion of older adults within societies is increasing and living longer. According to the World Health Organization (WHO), one billion people aged 60 + lived across the globe in 2019, which is expected to increase to 1.4 billion by 2030. Whilst an estimated 15% of older adults (aged 60 +) worldwide are living with a mental health disorder [31], this percentage does not capture undiagnosed lower levels of mental health and well-being, or fully account for stigma and other cultural barriers to acknowledging mental ill-health as defined in the global North.

The availability and accessibility of mental health care for the wider population tends to be limited in LMICs, with a lack of staff and support available [32]. Mental health problems are usually especially stigmatised in lower to middle income countries (LMICs) [11, 28] and thus low on the agenda, competing with more pressing needs, including food, living situation, and education. Given the high levels of stigma, even where resources do provide some forms of mental health support, people may feel unwilling to access available care [18], creating further barriers to fostering good mental health across the population. Stigma is also linked to a general reduced awareness of mental health, so that people who may benefit from care and support may not know where to access this or recognise this as the cause of their symptoms [23]. Considering the lack of mental health resources in LMICs, community-based approaches are especially relevant to support the population’s mental health and well-being. Overcoming for example the barriers of seeking out health care professionals by providing an accessible and acceptable form of support within their local community [15] is desirable.

Community-based psychosocial interventions for older adults have been found to effectively address mental health across various LMICs, including Thailand, Iran, China, and Malaysia [6]. No one form of intervention was found to be most effective, with educational, traditional structured therapy, social engagement, and exercise interventions, as well as multi-component interventions, shown to effectively reducing anxiety and depression and improving well-being [1, 17, 20, 29, 33]. However, older adults in different cultures and settings will experience different needs. This is particularly likely to be the case for older adults who have lived through extreme stressful life events, such as La Violencia, forced displacement, and other events and associated personal life impacts in Colombia. La Violencia was a particularly violent period in the history of the country, referring to a ten-year civil war between the Colombian Conservative and Liberal parties. These events are likely to have lasting impacts on how communities function and how older adults are integrated into society, as wider research into the impact of violence on the general population in Colombia has indicated [16, 24]. Thus, it is important to develop a culture- as well as needs-specific intervention for older adults in Colombia to support their mental well-being.

Colombia's internal armed conflict is one of the longest-running in the world. It is a public health problem, given the impact it has had on the country's living conditions, the way it alters life trajectories and its long-term effects, which cannot be solved individually, but requires state, social and community intervention to overcome the damage. As a result of the Peace Agreement in 2016, the Colombian Truth Commission has reported on the impact of the armed conflict from 1985 to 2018 [27]. In particular, the Truth Commission reports an estimate of over 450,000 fatalities, of which 80% were civilians who were not directly involved in the armed conflict. These fatalities, and the wider population, were subjected to severely physically and psychologically distressing and life-threatening events, including forced displacements (at least 8 million people), forced disappearances (110,000), kidnappings (50,000 people), and acts of sexual violence against women. Exposure to the armed conflict and associated violence has been linked to increased odds of experiencing mental health issues (Londono et al., 2012). This will have had a particular impact on the older adult population in Colombia, who have lived through the time of the armed conflict. With a lack of adequate mental health care available however, ways to support potential unmet mental health needs of older Colombians need to be explored.

The aim of this study was to feasibility test a co-produced intervention to improve mental well-being and reduce social isolation and loneliness among older adults residing in Turbo, Colombia, by increasing community integration.

## Methods

### Framework and underpinning theory

This intervention was developed according to the Medical Research Council framework for developing complex interventions [25]. Based on the core elements of the framework, we considered the context of the intervention by co-producing the intervention with older adults, carers, community leaders and organisations, thus also addressing the need for in-depth public and stakeholder involvement. We refined the intervention throughout the public consultation process, as well as several UK-Colombia team meetings, both virtually and face-to-face. The intervention is underpinned by Vygotsky’s social constructivism theory [26], which stipulates that learning takes place primarily in social and cultural settings. For this intervention, this means that learning about mental health and addressing mental health in older adults in Turbo, Colombia, is best addressed in a group setting with older adults who have all survived the armed conflict and other significant stressful life events (such as forced displacement, continued societal violence, and the coronavirus (COVID-19) pandemic).

### Participants and recruitment

Older adults aged 60 + living in San Martín, Turbo, Colombia, without moderate or severe cognitive impairment as assessed by the Montreal Cognitive Assessment (MoCA; [19]), were eligible to participate. Older adults were recruited by convenience sampling, using the snowball technique. San Martin is a neighbourhood where many older adults live, and they have formed community networks for many years. One psychologist and one community leader, who were both integrated in the community and were the local facilitators, sent out invitations via their established community networks.

Two face-to-face introductory sessions were held with those who accepted the invitation. The meetings were held in the space provided by one of the community leaders supporting the process. In these meetings, older adults were provided with information about the proposed intervention, and were invited to state the activities they wished to carry out during the intervention. Written informed consent was obtained from those who agreed to participate and the pre-test quantitative measures were completed at the session. Those who did not agree to participate in the evaluation were able to continue participating in the programmed activities without collecting their data. A subsample of participants from the intervention were invited to take part in a post-intervention semi-structured interview about their experiences.

## Intervention

### Development

This intervention was developed based on findings from a systematic review and meta-analysis [6], as well as an ongoing mixed-methods needs assessment of older adults residing in Turbo, Colombia, as part of the overall project [7].

A systematic review and meta-analysis of published community-based psychosocial interventions for older adults aged 60 + in LMICs [6] identified 40 studies from 12 countries, reporting on individual or multi-component interventions, including established forms of psychological therapy, exercise, education, and social engagement. Specifically, interventions included reminiscence therapy [29], yoga [20], ballroom dancing [33], educational training sessions [17], and intergenerational programmes [1]. Most interventions were effective in reducing levels of depression and anxiety, and improving well-being, whilst providing mixed levels of details about the actual interventions and its evaluation methods, thus highlighting the importance of well-documented procedures of future LMIC-based psychosocial interventions for older adults to increase replicability. Considering the variety of components found to be effective in the included interventions, there was no one single type of intervention approach found to be the most effective. This suggests that a combination of components, prioritised by the local population and stakeholders, resonating with their cultural norms and preferred activities, appears to be the most suitable approach for addressing older adults’ mental health and well-being within their own community. 

Findings on existing community-based psychosocial interventions for older adults in LMICs, and their efficacy, were complemented by strategic ongoing quantitative and qualitative needs assessments of the local population in Turbo, Colombia, as well as strategic co-production activities. The intervention has been conceptualised with the help of members of the public (older adults, family carers, community leaders, and community organisations). In particular, the team held detailed consultations with the administration of the municipality of Turbo, specifically with the Ministry of Health, and the mayor’s office whose staff are participating directly in the project through two psychologists from the District Secretary for health, protection and Social Welfare and the District’s Elderly Programme Department. The psychologists collected pre- and post-intervention data.

### Design of the intervention

Based on consulting residents and community leaders of the San Martin neighbourhood in Turbo, Colombia, we designed a three-month pilot intervention with two weekly sessions which varied in content based on four components, outlined below. Participants were able to attend as many or as few sessions at the community venue as preferred. As this was a feasibility study to explore whether the intervention would be accepted by the population group (older adults) and feasible to conduct, there was no control group.

### Content

The multi-component community-based intervention comprised four modules, comprising of 26 sessions, with two sessions provided each week for 12 weeks. The intervention included the following components split across four domains—social, educational, physical and peer support:

1. Social engagement
  The systematic review [6] suggested that social engagement interventions that have evidence of positively impacting on the quality of life and reducing feelings of loneliness, development of resilience and social support of older adults were: the intergenerational programmes reviewed in Iran [5] and Thailand [22] and the community-based day centres in India [10]. There were two elements to the social engagement domain:
  - *Intergenerational meetings:* Two 2-h meetings were held once a month (*n* = 6 meetings in total) in which older adults invited a younger member of their neighbourhood (school students, adult women and men, children). The activity was led by a psychologist, who provided a space to build answers around the question: *How do we age in Turbo and how would we like to age in the community?*
  - *Family meetings:* Meetings were held once a month and older adults invited one member of their family, children, grandchildren or cousins, with whom they wished to share this activity. The activities were led by a psychologist, who is the leader of the Elderly Programme of the District of Turbo. This activity encouraged listening to how older adults would like to participate in family life and how family members who accompany them can support them.
2. Educational interventions
  Educational interventions that have been shown to be effective include educational training in Iran [17], and health promotion programmes in China and Thailand [30, 34]. Techniques included lectures, presentations, use of printed material, self-learning, and discussion. For the intervention in the San Martin neighbourhood, we included two elements:
  - *Discussion on mental health and warning signs:* A two-hour meeting was provided once a month to open a community debate on mental health and its warning signs. The aim was to help residents of Turbo understand what mental health is, the warning signs to be aware of, and what kind of community-based actions could be taken to support those experiencing a mental health crisis. This activity was delivered by a mental health professional from the Mayor's Office of Turbo.
  - *Creation of protective mental health networks: referral routes to mental health services:* This activity was delivered once a month for two hours. It was carried out by the mental health professional assigned to the Urabá Region by the "Health for the Soul" Programme of the Sectional Health Secretariat of Antioquia. This activity consisted of training the community in the reduction of stigma towards mental illness, exploring ways to enhance and support parental involvement in the care of their children in partnership with older adult grandparents and towards their parents (now older adults) including warning signs of deterioration in mental health and options to access care for them from services (albeit limited).
3. Physical activities
  *Music therapy - Bullerengue evenings:* The community of San Martín is characterised by its ancestral knowledge of Bullerengue music. Bullerengue is a musical and dance practice of Afro-Colombian origin. It is an important cultural expression of Colombia. In San Martin there is one of the most representative leaders of Bullerengue in Turbo, a community leader, who learned this practice from his mother. Based on the ancestral knowledge they have and the Bullerengue classes they lead, the community leader runs the Bullerengue afternoons with participating older adults. The sessions lasted two hours, to the rhythm of the drums and the voices of the participants.
  - *Dance afternoons: *During the 2-hour gatherings, the older adult community attendees rotated the co-ordination and leadership of these sessions, including the music and dance content selected by the group. This was accompanied by healthy drinks and cooking traditional food, supporting healthy nutrition for the participants, who often lack access to balanced sufficient nutrition.
  - *Culinary memories of older adults: *As part of the Turbo tradition, cooking, fire, seafood, sweets, firewood, are common in the homes of families in this region. The participants in this co-production process have always alluded to the importance of food to ensure their well-being and mental health. Thus, the meetings helped rescue the ancestral culinary memories, sharing culinary and associated social traditions, connecting emotionally with the ancestors, the recipes they know and providing a space and opportunity for meeting around the kitchen, in the courtyards of the houses of the community of San Martin. The two-hour sessions focused on reminiscence about favourite foods and together choosing one recipe, to cook together at the following monthly session. Several dishes were made. The favourite foods were fish stew, coconut rice, bocachico tapado, fried fish, shrimps, stewed chicken, salted meat in widow, chicharrón with yucca and stewed meat. The food supplies required to cook the meal were provided by local donations and the project.
4. Peer support
  Peer support was promoted by community leaders. A listening activity was proposed by the leaders to promote understanding and learning among the older adults and their families to identify warning signs of mental health problems, ways to access care and emotional support, including District institutions such as the Hospital, Health Secretariat, Social Inclusion Secretariat, and the Family Police Station in these shared education sessions. The observers and leaders were accompanied by a psychologist and the leader of the programme ‘Adulto Mayor de Turbo’ (older adults in Turbo) to summarise discussions during the consultations, what issues arose and how to facilitate care routes for older adults.

### Delivery

Over twelve weeks, the intervention was held at a community centre in the San Martin neighbourhood of Turbo, with up to 25 older adults attending each session. The sessions were facilitated by the psychologist of the Mayor's Office who is in charge of the Senior Citizen program, and by a territorial liaison psychologist from the research team. The community leader who coordinates the Bullerengue groups in the District of Turbo participated in the cultural and music activities.

## Mixed-methods feasibility evaluation

### Quantitative

We collected baseline and post-intervention measures, including socio-demographic variables (at baseline only: age, gender, relationship status, educational level, ethnic group, location, socioeconomic background, and housing condition); the MoCA-B [13, 19] for overall cognitive functioning, the General Well-Being Index (WBI) [9], the social support scale (MOS scale) [8] and the scale of perception of social loneliness (ESTE II scale) [12]. The four instruments were applied by two trained psychologists.

### Qualitative

Within two weeks of the final intervention session, we conducted brief up to 15-min face-to-face interviews with a subsample of intervention participants who were interested in taking part. The semi-structured topic guide was produced among the Colombian and UK research team and asked attendees about their experiences of the intervention, its access, its effects, and any suggested changes or modifications to the intervention (see Appendix 1). Informed consent was obtained, and the interviews were conducted by three research team members (MIZ, EM, GS). The interviews were audio recorded and the audio files were transcribed anonymously, and translated into English by a trained translator. We analysed the transcripts using reflexive thematic analysis [4] using the 6-step model, underpinned by social constructivism theory [26], with each transcript coded in Spanish and in English and thus by two research team members. Once all transcripts were coded and researchers (TC, EM) had individual generated codes, the team met virtually to discuss the quotes and jointly cluster these into emerging themes.

### Ethics

Ethical approval was sought from the Research Ethics Committee of the National Faculty of Public Health, University of Antioquia, prior to the intervention and data collection [REF: 21,030,002–0019-2020].

## Results

### Participant demographics

Eighteen older adults participated in the baseline assessment, and thirteen older adults completed both pre- and post-intervention measures. Demographic characteristics are provided in Table 1.

**Table 1:** Demographic characteristics

	***N* = 18**	***N* = 13**
**Demographic**	**Mean (SD) [Range]**	**Mean (SD) [Range]**
Age	71.2 (5.5) [61–84]	71.6 (5.8) [62–84]
Number of people living in same household	4.4 (2.1) [2–9]	4.5 (2.1) [1–8]
	**N(%)**	**N(%)**
Gender		
Female	16 (88.9%)	11 (84.6%)
Male	2 (11.1%)	2 (15.4%)
Relationship status		
Single	11 (61.1%)	5 (38.4%)
Widowed	3 (16.7%)	2 (15.4%)
Separated	2 (11.1%)	4 (30.8%)
Married	2 (11.1%)	2 (15.4%)
Education		
No school	2 (11.1%)	2 (15.4%)
Incomplete primary school	8 (44.4%)	6 (46.2%)
Primary school	2 (11.1%)	2 (15.4%)
Secondary school	4 (22.2%)	2 (15.4%)
Incomplete		
Secondary school	2 (11.1%)	1 (7.7%)
Complete		
Caring responsibilities		
Yes	12 (67%)	7 (54%)
No	6 (33%)	6 (46%)

### Attendance rates

Thirteen of 18 participants completed the intervention, highlighting a completion rate of 72%. Those who dropped out of the intervention did so because unfortunately they became too ill to participate, had increased caring duties or were unable to find the time to attend, and had to move to family outside of Turbo to meet their own increased care needs. Of the 18 participants, the majority attended between 90–100% of the 26 sessions. Four attended fewer than 13 sessions, with three and one attending between 50–60% and 70–80% of the sessions, respectively. A full overview of attendance rates is provided in Table 2.

**Table 2:** Percentage of participation and attendance

Percentage of participation	n	%
90–100%	10	55.5
80–90%	0	0
70–80%	1	5.6
60–70%	0	0
50–60%	3	16.6
40–50%	2	11.1
30–40%	1	5.6
< 30%	1	5.6
Total	18	100

A total of 26 sessions (100%) were provided

### Qualitative evaluation

A total of 12 interviews were conducted. Our sample largely consisted of women (women: *n* = 9 (75%); men: *n* = 3 (25%)). The inductive thematic analysis generated the following themes: ‘The intervention: views and experiences’ and ‘wellbeing: short and longer-term impact.’ Quotes are included in Table 3, organised by themes and subthemes.

**Table 3:** Quotes by themes and subthemes

Theme	Quotes
**Theme 1: The intervention: views and experiences**	
*Appetite and (non)attendance*	‘No… straight away, I said: 'I'll go and see what it's like. I'll go along, and if I don't like it then I won't go back.' **Man, P03** ‘She said to me: “Mama, we’re going to put on some activities. If you want to get involved… You’d also be part of the programme. Though you might forget, or just want to stay watching telly.” I don’t know… It was like she was telling me off! I said to her: “no, I’ll go. I’ll go.” I got myself out of the boring routine and had those two days a week [intervention]. They were great. Really great.’ **Woman, P12** ‘Because some of the, thought that this was a political thing. A lot of them told me that they weren't going to come along because they don't want to get involved in politics. I said 'no way! It's nothing political', you know? I told that is wasn’t anything to do with politics. After that, a few of them did come along when I asked them to. But there were some who still didn't.’ **Woman, P02** ‘There aren't many men in the club, but then there aren't many men in the elderly population as a whole. There are more women than men.’ **Woman, P02** ‘They [men] didn't come because they hadn't heard about it, or they didn't know.’ **Man, P03** ‘Men are less outgoing… more shy, from what I can tell. There aren't many men who go to church, either… I don't know. I’ve seen them [men] meeting up in little groups in the park, in certain areas. I don't know why they wouldn't want to come to something like this to relax and to chat with other people.’ **Woman, P09** ‘When I got to the spot were [organiser's name]'s dad was, he told me: ' Ah! They’ve left. They've gone to the beach' So I just turned around; there wasn't anyone who could take me. So, I didn't go.’ (**Woman, P06**)
*Acceptability*	‘The way those girls who organised everything to know me… How those girls treated me. They were like “Oh, here comes another one”. I don't know what it was. It was, like, a feeling of being… there was no discrimination. Once you were on the programme you were accepted, here. I liked that about them. How they treated us.’ (**Woman, P07**) ‘We went in the care [home]; they picked us up from here and took us there. Then they brought us back, to the same place. Then, from here, everyone made their own way home. It worked well.’ **Man, P03** ‘There was one session in which we all reminisced about our childhood. Yeah. I really liked thinking about when we were young; the time we spent with our parents.’ **Woman P12** The least…. well, none of them. We had fun in all of them. The lady in charge of… Of the Bullerengue [traditional dance] … There was always somethings or other [that we could do]. **Woman, P01** ‘I wanted the meetings to carry on, though. That would be my suggestion.’ **Man, P03** ‘It’d be really nice if it carried on. I’d like that, especially, because it was a nice way to spend our evenings.’ **Woman, P10** ‘You have to make a lot of sacrifices to come here, but it's really good.’ **Woman, P07**
**Theme 2: Wellbeing: shorter and longer-lasting impact**	
*Forging friendships*	‘Sometimes you can feel very lonely, as you always follow the same routine – just talking to the same people at home all the time.’ **Woman P11** ‘So, sometimes when I'm at home, I feel like I'm on my own. I try to get out of the house. I don't really like being at home on my own.’ **Man, P08** ‘You can just let yourself go. No one bothers to say anything because we're all in the same boat.’ **Woman, P02** ‘…we told stories, we spent time remembering the war, and remembering the time when there was not war; we used to just gossip.’ **Woman, P06** **‘**When you're going through something, and you can't… You can't express it; you can't tell anyone. You just have to swallow it. You need to tell those things to, and then you can get a break from it all’** Woman P07** ‘Most of all I think that I have more friends now than I used to. I’d not spent much time with the people who came here, and now they’re my friends. It’s not like it was before.’ **Woman, P12**
*‘Keeping a busy mind’*	‘Those things are great because they keep your mind occupied. When you’re just sitting around at home, you end up with different problems. But, on the other hand, as soon as you’re here you don’t think about those things. You’re in the moment. I really liked… I really liked the programme’ **Woman, P10** ‘It [feeling of happiness] lasted for three, or four days. Depending on… well, we used to come on a Wednesday and a Friday, so we would stock up [on that feeling]. After a few days, it would be time to come back.’ **Woman, P06** ‘There were times when I thought I couldn't do something, but then I realised that wasn't true. People think that, when you get older, you're not capable of doing things. But that's not right. I realised, while I was here, that I still have a lot of mental skills.’ **Woman, P07**
*(Re)building intergenerational connections*	'Children can be so, so cruel; there are times when I'm with them and I may as well not exist. That's what they're like. At least they’re at my house.’ **Woman, P01** ‘The kids were very polite; they treated us well, and were keen to hear what we had to say.’ **Woman, P09** ‘For me, [it helped] in the sense that people at the centre spoke a lot about ageing, and how their grandchildren treated them. It helped me because I always used to come with my grandson, like I told you. He learnt so much during that time, about respect. Because he was one of the most… well, you know what kids can be like…’ **Woman, P07**

### Theme 1: The intervention: views and experiences

Overall, the intervention observed high attendance and was enjoyed by all participants interviewed. This theme is divided into the follow sub-themes: ‘appetite and (non)attendance’ and ‘acceptability.’

#### Appetite and (non)attendance

When invited to participate in the intervention, most participants agreed without hesitation. Family members of most participants were supportive of their participation, with one participant reporting she was strongly encouraged to attended by her daughter.

Some participants spoke about the intervention to their friends who might be interested in joining. Mixed results were observed. One participant reported that his friend ‘*wasn’t bothered*’ (Man, P03) and one participant reported that their friends would be interested, but declined joining as they were unsure whether the intervention was open to people residing in different neighbourhoods. Moreover, one participant reported that some of their friends declined the invitation due to mis-perceiving the intervention to be associated with politics. Participants named the intervention ‘Nueva Esperanza’ (new hope) and, when asked, a participant suggested that including the University of Antioquia within the name of the intervention may prevent future misunderstandings.

Overall, few men participated in the intervention. When asked why, many participants were unsure, with some suggesting that the low number of older men in general, poor awareness of the intervention and/or shyness could explain the limited uptake.

In general, participants attended most of the sessions. Reasons for nonattendance included illness, medical appointments, child minding responsibilities, forgetting and/or arriving late. One participant reported *‘…losing sales because of this! [laughing]*’ (Woman, P10), but deemed it more important to attend the sessions.

#### Acceptability

Most participants reported that the intervention was well planned, felt accepted and that they enjoyed attending the sessions. Following the first session, one participant thought to herself ‘*Ah, I've been looking for something like this*’ (Woman, P05).

Participants found it challenging to identify a favourite and least favourite session, as they enjoyed most sessions. Preferred sessions included cooking and sharing a meal, trips to the beach, dancing, reminiscing about the past, spending time with children and visiting the University of Antioquia.

When asked how the intervention could be improved, all participants expressed the desire for the sessions to continue. The input from the study team was deemed important ‘*… because without them we can’t start it up again’* (Woman, P07). Some participants suggested that, going forwards, sessions could include additional excursions, craft making, teaching literacy skills and increasing the number of sessions per week.

Regarding the frequency of sessions per week, participants’ views were mixed. While some favoured meeting more frequently, other participants expressed concern due to competing priorities, including child minding responsibilities and employment.

### Theme 2: Wellbeing: shorter and longer-lasting impact

All participants reported that participating in the intervention benefitted their wellbeing, in some form, with one participant expressing that ‘*I probably needed them [sessions] earlier in my life*.’ (Woman, P11). Benefits to participants’ wellbeing were largely experienced while attending sessions, however, some seemed to be longer-lasting. This theme is divided into the following sub-themes: ‘forging friendships’, ‘keeping a busy mind’ and ‘(re)building intergenerational connections.’

#### Forging friendships

Prior to the intervention, many participants reported feeling lonely. Some participants had little interaction with their family members and/or many spent most of their time at home. While most participants came from the same neighbourhood, many were not overly acquainted with one another. Sessions helped to break ‘*down a wall*’ (Woman, P07) to forge a trusting and supportive camaraderie among participants, which seemed to serve multiple benefits.

Spanning from sharing moments of laugher, letting ‘*off some steam*’ (Woman, P09) to disclosing traumatic life events, socialising with other participants helped to combat feelings of loneliness and provided a safe environment for difficult stories to be told. Traumatic life events included an incidence of domestic violence, loss of close family members and/or experiences relating to the internal armed conflict in Colombia.

Many had ‘*a great time just talking*’ (Woman, P09) and socialising helped some participants feel less stressed and/or worried. Forged friendships endured beyond the intervention, with several participants remaining in contact with one another, which took form in spontaneous ‘catch-ups’ while seeing one another in the neighbourhood and/or organised gatherings.

#### ‘Keeping a busy mind’

The sessions served as a distraction to many participants, granting them a couple of hours away from their worries, low moods and helped to manage stress levels.

Nevertheless, feelings of happiness and/or relaxation did not seem to last, with some participants reporting negative feelings to return in the same or, within the following days. One participant reported that their family members found them more relaxed and less angry since attending the intervention.

Prior to the intervention, some participants reported feeling forgetful. These included arriving to the shops and forgetting what they had planned to purchase and forgetting what they intended to say. Following the intervention, many participants reported feeling less forgetful and more ‘*alert*’ (Woman, P05). Moreover, some children taught the older adults some ad hoc skills in the intergenerational sessions, which were not pre-planed but based on their interactions within the sessions. This included how to use mobile phones. Many participants appreciated the opportunity to learn new skills. One participant surprised themselves that they could learn new skills at their age and one participant reported that they had forgotten, at the time of interview, the skills they were taught.

#### (Re)building intergenerational connections

Some participants commented on the lack respect received from younger generations. As part of the intervention, children from a school were invited to teach participants how to use mobile phones and to listen to stories about Colombia’s past. Participants enjoyed spending time with the children, reporting that they were ‘*really respectful*’ (Woman, P01), ‘*very caring’* (Woman, P02*)* and ‘*lovely*’ (Woman, P11). One participant seemed surprised that the children were willing to spend time with them and one participant was upset when the children left.

Moreover, some participants invited their grandchildren to activities as part of the intervention. One participant reported that by learning about older adults’ experiences, her grandson treated her with more respect.

## Discussion

This co-produced and evidence-based intervention is one of the first to provide a community-based approach for older adults’ mental health and well-being in a conflict affected country. This study showed that the co-development of the intervention and its evaluation were feasible to conduct, with high levels of attendance rates from the older adults, and particularly a keen interest in continuing the intervention after it terminated, as evidenced in the qualitative findings.

This intervention was co-produced from the outset with local residents, ensuring that the intervention met the needs and interests of potential participants as well as enabling shared ownership. This strong element of co-production may also have contributed to the high attendance rates, with illness and increased caring duties reported to be the sole reasons for discontinuation. Whilst the value of co-production in shaping mental health interventions regardless of setting is well-evidenced (i.e. [3, 21]), co-producing interventions in LMIC settings is little reported on, as a recent systematic review on community-based mental health interventions for older adults has shown [6]. Thus, this study contributes novel evidence as to the potential value and impact of involving target population groups and other relevant stakeholders in the intervention design from the very beginning.

## Limitations

Whilst the intervention showed high acceptability in the older adult population, outcome measures were not found to be suitable enough for the population group. Outcome measures were selected based on their cultural adaptations. However, the study sample was relatively illiterate, which is common for populations in Colombia, and other Latin American countries and LMICs more broadly [2, 14]. This was not considered a limitation but the fact that measures were not culturally and educationally sensitive enough was a limitation of the existing tools, not this study. Thus, the next stage in this intervention will require further modified and culturally and educationally adapted outcome measures to capture the impact of the intervention on mental well-being, quality of life, and loneliness more adequately.

## Conclusions

This co-produced community-based mental health intervention has the potential to improve the well-being of older adults in Colombia and other LMICs. In a country where support systems for older adults are often lacking, providing a multi-component intervention to support older adults’ mental well-being which is developed by and embedded within the local community can provide an important gateway to supporting older adults live well. The next step will be to conduct the intervention across Colombia, in not only rural, but also urban and peri-urban settings, and test its effectiveness with a larger sample size. This intervention has the potential to be adapted to different cultures also, offering a feasible and low-cost intervention to address older adults’ mental health.

## Supplementary Information

Supplementary Material 1.

## Data Availability

Data may be shared upon reasonable request if contacting the contact author.
