# Supplementary material for: Addressing unmet mental health needs of older adults in Turbo, Colombia: a multi-component psychosocial intervention feasibility study
Source: Int J Equity Health. 2025 Jan 20;24:21. doi: 10.1186/s12939-025-02381-x (PMC11748288; doi:10.1186/s12939-025-02381-x)
Supplement: Supplementary file 1 — Supplementary Material 1. [file 12939_2025_2381_MOESM1_ESM.docx]

**Appendix 1 – Semi-structured Topic Guide for Participants**

**Introduction**

We are interested in your experience of the psychosocial intervention in Barrio San Martín, the feelings and meanings you have had in participating in the activities. There are no right or wrong answers, we are interested in your individual thoughts and experiences. Please share with us as much as you feel comfortable.

**Questions**

- How did you find out about the meetings?
- Where you able to attend all meetings? If not, what were the reasons for not attending every meeting?
- How did you feel about the activities that took place at the meetings?
- Did you notice any positive, or negative, changes as a result of attending the meetings?
- What impacts have the activities had on your life?
- Of the meetings you attended, which did you like the most, which did you like the least? Why?
- What other activities would you like to see at the meetings? And are there any other suggestions you may have for future interventions of this kind?
